# Identification and Confirmation of the miR-30 Family as a Potential Central Player in Tobacco-Related Head and Neck Squamous Cell Carcinoma

**DOI:** 10.3389/fonc.2021.616372

**Published:** 2021-07-13

**Authors:** Tingting Zhang, Xueqin Zhu, Qiang Sun, Xing Qin, Zhen Zhang, Yuanyong Feng, Ming Yan, Wantao Chen

**Affiliations:** ^1^ Department of Oral Maxillofacial Head and Neck Oncology, Shanghai Ninth People’s Hospital, Shanghai Jiao Tong University School of Medicine, College of Stomatology, Shanghai Jiao Tong University, Shanghai, China; ^2^ National Center for Stomatology, National Clinical Research Center for Oral Diseases, Shanghai Key Laboratory of Stomatology, Shanghai, China; ^3^ Department of Oral and Maxillofacial Surgery, Stomatological Hospital, Tianjin Medical University, Tianjin, China; ^4^ Department of Oral and Maxillofacial Surgery, The First Affiliated Hospital of Zhengzhou University, Zhengzhou, China; ^5^ Department of Oral and Maxillofacial Surgery, the Affiliated Hospital of Qingdao University, Qingdao, China

**Keywords:** head and neck squamous cell carcinoma, miR-30, 4-nitroquinoline 1-oxide, tobacco smoke, KRAS

## Abstract

Constituents of tobacco that can cause DNA adduct formation and oxidative stress are implicated in the development of head and neck squamous cell carcinoma (HNSCC). However, there are few studies on the mechanism(s) that underlie tobacco-associated HNSCC. Here, we used a model in which tumors were induced in rats using 4-nitroquinoline 1-oxide (4NQO), which mimicked tobacco-related HNSCC, and analyzed the expression profiles of microRNAs and mRNAs. Our results indicated that 57 miRNAs and 474 mRNA/EST transcripts exhibited differential expression profiles between tumor and normal tongue tissues. In tumor tissue, the expression levels of rno-miR-30 family members (rno-miR-30a, rno-miR-30a-3p, rno-miR-30b-5p, rno-miR-30c, rno-miR-30d, rno-miR-30e and rno-miR-30e-3p) were only 8% to 37% of those in the control group. The GO terms enrichment analysis of the differentially expressed miRNAs indicated that oxidation reduction was the most enriched process. Low expression of miR-30 family members in human HNSCC cell lines and tissues was validated by qPCR. The results revealed that the expression of miR-30b-5p and miR-30e-5p was significantly decreased in the TCGA HNSCC dataset and validation datasets, and this decrease in expression further distinguishes HNSCC associated with tobacco use from other subtypes of HNSCC. CCK8, colony formation, transwell migration and HNSCC xenograft tumor assays indicated that miR-30b-5p or miR-30e-5p inhibited proliferation, migration and invasion *in vitro*, and miR-30b-5p suppressed tumor growth *in vivo*. Moreover, we uncovered that KRAS might be the potential target gene of miR-30e-5p or miR-30b-5p. Thus, our data clearly showed that decreased expression of miR-30e-5p or miR-30b-5p may play a crucial role in cancer development, especially that of tobacco-induced HNSCC, and may be a novel candidate biomarker and target for this HNSCC subtype.

## Introduction

Head and neck squamous cell carcinoma (HNSCC) is one of the most common malignant tumors worldwide, accounting for approximately 3.8% of all malignant cancers (1, 2). The consumption of tobacco is an established major etiological factor in the development of HNSCC (3). In the Indian subcontinent, HNSCC is the single most common malignancy, and it is mainly caused by the use of chewing tobacco and related products (4). It was reported that smoking is responsible for most deaths from HNSCC in high-income countries (5). Over the past few decades, the HNSCC incidence rate has increased among both males and females in several countries in Eastern and Northern Europe and among females in Southern and Western Europe, which largely reflects the ongoing tobacco epidemic (6, 7). In many other more developed countries, as tobacco use has decreased, the HNSCC incidence rate has begun to decline in both males and females (7).

Over 60 chemicals found in tobacco products, such as dibenz[a,h]anthracene, benzo-(a)-pyrene, 4-aminobiphenyl, acetaldehyde, N-nitrosamines, catechol and benzene, have been shown to be carcinogenic (8, 9). Carcinogens can be metabolized by cytochrome P450 enzymes into water-soluble forms, generating DNA adducts (10). These adducts usually cause sequence variations in DNA. If not repaired correctly, these lesions lead to tumor formation (11). Despite treatment advances, the 5-year relative survival rate still has not improved significantly (12), which can be attributed primarily to diagnosis at an advanced stage characterized by invasion and metastasis to the lymph nodes and remote organs (13). Identification of predictive biomarkers for diagnosing HNSCC at earlier stages would be beneficial for patient survival. MicroRNAs (miRNAs) are a class of small noncoding RNAs that play an important role in regulating gene function through targeting mRNAs for translational repression or degradation (14). Deregulation of miRNA expression contributes to aberrant expression of mRNAs that mediate the complex malignant phenotypes of cancers. Changes in a number of miRNAs and genes that are required for the occurrence of HNSCC have already been identified, including increased expression of miR-21-5p and miR-31-5p and reduced expression of miR-375 and miR-100 family members. However, a large gap in our understanding of how the different miRNAs and mRNAs expressed during HNSCC development relate to exposure to tobacco remains.

4NQO is a synthetic, water-soluble carcinogen that can result in histological and molecular alterations very similar to those found in human tobacco-related oral carcinogenesis by promoting DNA adduct formation, A-G nucleotide substitution and intracellular oxidative stress (15, 16). The murine model of 4-nitroquinoline 1-oxide (4NQO)-induced oral carcinogenesis has been used extensively to identify how chronic tobacco abuse contributes to human HNSCC and how therapeutic treatments can alleviate or prevent these malignancies (16–18).

Determining the relationships among the expression levels of the various microRNAs and mRNAs could be useful for identifying diagnostic markers or therapeutic interventions. Most microRNAome and transcriptome data were derived from separate studies on oral cancer that did not address risk factors. In this study, we used the 4NQO-induced carcinogenesis model to mimic human tobacco abuse to identify the network of miRNAs and their predicted target mRNAs involved in HNSCC. Remarkably, we revealed downregulated expression rno-miR-30 family members which targeted several classical oncogenes, such as KRAS, centering on cell growth, proliferation and invasion, may be important in initiating the development of HNSCC, especially tobacco-related tumors.

## Material and Methods

### Tumor Induction

**Figure 1:**
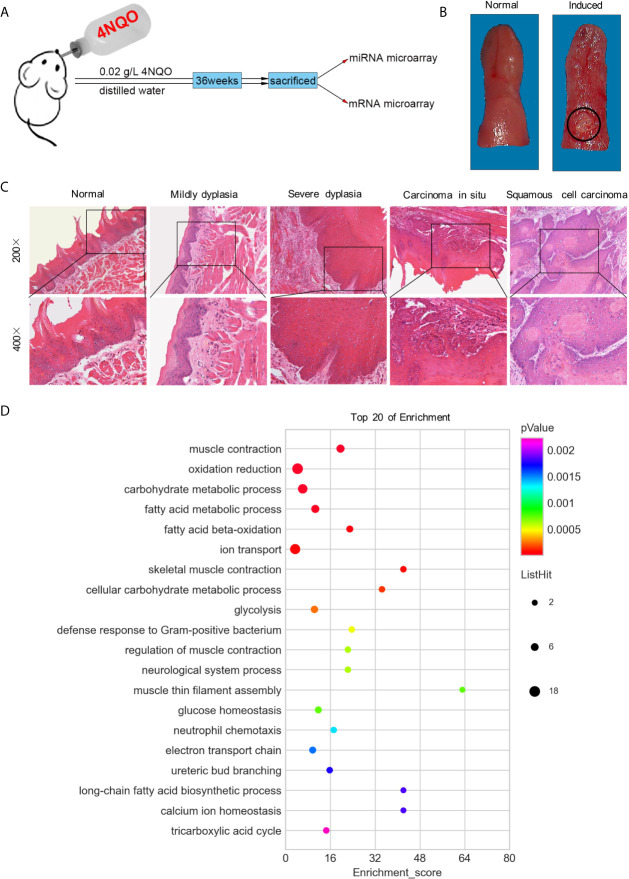
Both macroscopic and microscopic changes in SD rats’ tongue mucosa. **(A)** Experimental scheme of SD rats induced by 4NQO for 36 weeks. **(B)** Photograph of normal tongue (left) and exophytic neoplasm on tongue induced by 4NQO water for 36 weeks (right). **(C)** H&E staining showed the pathological changes of rat tongues base at different stages. **(D)** GO analysis of top 20 of functional enrichment based on mRNA microarray.

The animal model was established as previously described (19). Briefly, 40 male Sprague-Dawley (SD) rats, aged 6-7 weeks, were randomly divided into two groups. In the experimental group (n=20), the rats were fed daily with dissolved 4NQO (Sigma, USA) at a concentration of 0.02 g/L, whereas the control group (n=20) were fed distilled water only. At the end of the 36^th^ week, all rats were sacrificed (**Figure 1A**). Tumors and normal tongue tissues were collected from the experimental and control groups, respectively; and separated into two parts: one was set aside for histopathological examination, and the other was instantly placed into liquid nitrogen and then stored at -80°C for RNA extraction.

### Total RNA Isolation and miRNA/mRNA Microarray

Total RNA was extracted from 7 tongue SCC tumors and 7 normal tissues using mirVana RNA Isolation Kit (Applied Bio-system p/n AM1556) following homogenization. 7 rats per group were pooled for miRNA microarray, and 5 normal tongue tissue and 6 tongue SCC tumors were carried in mRNA microarrays. RNA labeling, hybridization and scanning were performed by ShanghaiBio Corporation using a Rat miRNA microarray (Agilent, G4473A). The GeneSpring GX software (Agilent Technologies) was used to analyze the microarray data. Preparation of antisense RNA, hybridization, staining, and scanning of the Rat Genome U34 Array (Affymetrix Inc.) was performed according to the Affymetrix standard protocol. Data normalization and analysis were performed with Microarray Suite version 5.0. Differential miRNA/mRNA expression was determined as at least a 2-fold difference between the experimental and control groups.

### miRNA/mRNA qRT-PCR for Validation

MiR-30c, miR-30d and miR-30e-3p were chosen as the candidate miRNAs for microarray data validation. U6 RNA was chosen as an internal control. The 20 μL reverse transcriptase reactions contained 1 μg of RNA and were completed using Mircute enhanced miRNA cDNA first strand synthesis Kit (TIANGEN, China). Real-time PCR was performed using Mircute enhanced miRNA fluorescence quantitative detection kit (TIANGEN, China). The genes of Ubadc1, Ldhb, and Cabc1 were selected to validate the microarray results by Quant one step qRT-PCR Kit (SYBR Green) (TIANGEN, China). β-actin was chosen as an internal control. The sequences of the PCR primers (synthesized by Sangon Biotech Co, China) were shown in **Table S1**.

### Gene Ontology (GO) Analysis of rno-microRNA Target Genes

GO analysis was applied to analyze the main function of the differentially expressed microRNA targets according to the Gene Ontology database (http://www.geneontology.org/) (20). Fisher’s exact test and χ2 test were used to classify the GO categories, and the false discovery rate (FDR) was calculated to correct the *p*-value. GOs that had a *p*-value of <0.05 and a FDR of < 0.05 were chosen for further study.

### MiRNA-Gene-Network

The miRNA-gene-network was built according to the interactions of miRNA and genes in the Sanger miRNA database. The adjacency matrix of miRNAs and genes, A=[ai,j], is made by the attribute relationships among the genes and miRNAs, and ai,j represents the relative weight of gene i and miRNA j. Degree indicates the contribution of one miRNA to the nearby genes or the contribution of one gene to the nearby miRNAs.

### MiRNA-GO-Network

A miRNA-go-network was built according to the relationship between significant GOs and genes as well as the relationships among miRNAs and genes. The adjacency matrix of microRNA and genes, A=[ai,j], was made by the attribute relationships among GOs and miRNA, and ai,j represented the relative weight of GO i and miRNA j. Degree indicated the contribution of one miRNA to the nearby GOs or the contribution of one GO to the nearby miRNAs.

### SD Rat Cell Culture

The SD rat SCC cell lines Rca-T (21) and Rca-B (22) were cultured in DMEM (Gibco, USA) supplemented with 10% FBS, 100 units/mL penicillin and 100 mg/mL streptomycin at 37°C in a humidified atmosphere of 5% CO_2_. The primary culture came from buccal mucosa of male Sprague-Dawley (SD) rats, aged 4-5 weeks. The tissue was cut to 0.2 cm×0.2 cm pieces, then they were incubated in 5 mL 0.8% dispase II (Roche, Switzerland) solution at 4°C overnight. Epithelium was isolated from basal lamina, and put into 3 mL 0.25% trypsin for 5 minutes. The suspension was centrifuged in low speed and supernatant was decanted. The deposition was resuspended and incubated with Defined Keratinocyte SFM (Gibco, USA) in a 6 cm dish.

### Cell Culture

Human HNSCC cell lines HN4, HN6, HN30 and SCC9 were obtained from University of Maryland Baltimore School of Dentistry and ATCC, respectively. HN4, HN6, and HN30 cells were maintained in DMEM (Gibco, USA) supplemented with 10% FBS (Gibco, USA), 50 U/mL penicillin, and 50 μg/mL streptomycin. SCC9 cells were maintained in DMEM/F12 (proportion: DMEM with F12 was 1:1, Gibco, USA) supplemented with 10% FBS, 50 U/mL penicillin, and 50 μg/mL streptomycin. HEK-293 cells were incubated in DMEM supplemented with 10% FBS, 50 U/mL penicillin, and 50 μg/mL streptomycin. All the cells were incubated at 37°C in a humidified atmosphere with 5% CO_2_. Human normal oral mucosa came from the local normal mucosal tissue removed during the extraction of impacted teeth, and the mucosal tissue was in a healthy state without inflammation. The procedure for the culture of primary cells of human oral mucosa is basically the same as that of primary cells of oral mucosa of SD rats.

### Patients and Clinical Specimens

Paired HNSCC and adjacent noncancerous tissues were obtained from 32 primary HNSCC patients at the Department of Oral Surgery, Ninth People’s Hospital, Shanghai Jiao Tong University School of Medicine, Shanghai, P. R. China. The experiment has been approved by the Ethics Committee of Ninth People’s Hospital Affiliated to Shanghai Jiao Tong University School of Medicine. These tissues were flash-frozen in liquid nitrogen immediately after resection and stored at -80°C. None of the patients had received neoadjuvant chemo- or radio-therapy before surgery.

### Real Time PCR

Total RNA was isolated from Rca-B, Rca-T, HN4, HN6, HN30, SCC9, human oral mucosal epithelial cells using the TRIZOL Reagent (Invitrogen, USA). Total RNA was isolated from 32 paired HNSCC and adjacent noncancerous tissues using the TRIZOL Reagent (Invitrogen, USA). MiR-30 family was detected by qRT-PCR. The procedure was the same as above. The sequences of the PCR primers (synthesized by Sangon Biotech Co, China) were shown in **Table S1**.

### Cell Transfection

SCC9 or HN4 cells at a density of 1×10^5^ per well were seeded into a 6-well plate and incubated overnight respectively, then transfected with mature miR-30b-5p mimic, miR-30e-5p mimic, miR-30b-5p inhibitor, miR-30e-5p inhibitor or negative control (Riobo Bio, China) using Lipofectamine 2000 (Invitrogen, USA) according to the manufacturer’s instructions. Total cell RNA was extracted by addition of 1 mL TRIZOL reagent (Invitrogen, USA) to the cells in each well according to the manufacturer’s protocol and used for qRT-PCR to detect the expression of miR-30b-5p and miR-30e-5p. The procedure is the same as above.

### Cell Proliferation Assay

The transfected SCC9 or HN4 cells were plated in 96-well plates at 1000 cells per well and incubated at 37°C. CCK8 assay was performed at 0, 1, 2, 3, 4, 5, 6 day, respectively. A 10 μL of CCK8 solution (DOJINDO, Japan) was added and incubated for 2 h at 37°C, then optical density (OD) was measured at 450 nm in a microplate spectrophotometer.

### Colony Formation Assay

The transfected SCC9 or HN4 cells at 500 cells per well were seeded into 6-well plate and incubated at 37°C for 10 days, colonies were fixed and stained with 0.1% crystal violet for 1 h. Then the clones were counted and normalized to the control group under a microscope.

### Transwell Migration Assay

The transwell migration was performed using 24-well transwell chambers (8 μm, Millipore, USA). After transfection, 2×10^4^ SCC9 or HN4 cells were resuspended in serum-free medium and seeded into the upper chambers. We added 0.6 mL medium containing 20% FBS to the bottom chambers. Following a 30 h incubation, cells on the upper surface of the membrane were removed using cotton swabs, and the invaded cells were fixed with 4% paraformaldehyde for 15 min, stained with 0.1% crystal violet for 1 h, and imaged using microscope. The areas of crystal violet were calculated by Image J 1.53 c (National Institutes of Health, USA).

### Wound Healing Assay

About 1.5×10^6^ transfected SCC9 or HN4 cells were seeded into 6-well plate and incubated at 37°C and grew until 100% confluence. Sterilized one-milliliter pipette tip was used to generate wounding across the cell monolayer, and the debris was washed with PBS. Migration of cells into the wound was then observed under inverted microscope at 0 and 12 hours.

### Luciferase Reporter Assays

Vectors encoding the wild-type or mutant 3’UTR of KRAS, cloned behind Renilla luciferase, were constructed by Genomeditech (China). HEK-293 cells were seeded 24-well plates, the miRNA mimics and vector were transfected with HG transgene reagent (Genomeditech, China) when the cell density up to 70%. The cells were lysed with cell lysing buffer and luciferase activity was detected using the Renilla-Glo^®^ Luciferase Assay System (Promega, USA) following the manufacturer’s instructions. All measurements represent the mean of 3 replicates in each experimental condition.

### Western Blot

HN4 cells were transfected with miRNAs described above and then lysed by RIPA lysis buffer (Roche, Switzerland). The concentration of protein was determined using a BCA Protein Assay (Thermo Scientific, USA). Total protein at 30 μg was subjected to SDS-PAGE on a 4-12% gradient Bis-Tris gel (Invitrogen, USA). Protein was transferred to a 0.22 μm PVDF membrane which blocked with 5% skim milk. Primary antibodies used for probing are listed below. Secondary antibodies labelled with HRP (Beyotime, China) were used for detection at a dilution of 1:1000 and detected using Chemiluminescent HRP Substrate (Merck Millipore, USA), and signals were captured and observed using an Amersham Imager 600 (GE, USA). Primary antibodies: KRAS (1:5000, rabbit, Proteintech, China), GAPDH (1:10000, mouse, Proteintech, China).

### Nude Mouse Tumorigenicity Assay

The nude mouse tumorigenicity model was constructed with 5-week-old male BALB/c nude mice. The mice were purchased from the Shanghai Sippr-BK laboratory animal Co. Ltd. and were housed in pathogen-free cages (n = 3/cage) with a controlled light/dark cycle (12/12 h) at a temperature of 21°C. The animal use protocol was approved by the Institutional Animal Care Committee and all procedures conformed to Animal Studies Committee-approved protocols. A total of 1×10^6^ HN4 cells in 100 μL serum-free DMEM were injected into bilateral hip. A week later, the nude mice developed tumors in their buttocks. The mice were divided into 6 groups according to the miRNA injected: NC agomir, miR-30b-5p agomir, miR-30e-5p agomir, NC antagomir, miR-30b-5p antagomir, miR-30e-5p antagomir (Riobo Bio, China) (**Figure 6A**). Then miR-30b-5p or miR-30e-5p agomir were injected around the tumor at a dose of 5 nmol/tumor, twice a week for 4 times. MiR-30b-5p or miR-30e-5p antagomir was injected around the tumor at a dose of 10 nmol/tumor, twice a week for 4 times. The body weight and tumor size were measured regularly during the experiment. The mice were sacrificed at 12^th^ day and tumors were dissected, then the tumor fixed with formalin for further hematoxylin and eosin (H&E) staining and immunohistochemistry (IHC) analysis.

### IHC

Excised tumor tissues were fixed in 4% paraformaldehyde, dehydrated, paraffin-embedded, and cut into 5 μm sections. The sections were analyzed using primary antibodies against KRAS (1:800, rabbit, Proteintech, China), Ki67 (1:2000, rabbit, Proteintech, China) and a biotin-conjugated goat anti-rabbit polyclonal antibody (1:50; ZSGB-BIO, China) as the secondary antibody. Images were obtained by light microscopy (Olympus, Japan) at 400× magnification.

### Analysis of TCGA Data

The HNSCC miRNA expression profile from TCGA was download through GDC API, the expression matrix was tidied by TCGA biolinks (23, 24). The putative biological role of the targets genes was performed by clusterProfiler that described by Yu et al. (25).

### Functional Enrichment Analysis

The target genes of selected miRNA were predicted with the ENCORI (The Encyclopedia of RNA Interactomes) method. Potential target genes of miR-30b-5p or miR-30e-5p were predicted by ENCORI (http://starbase.sysu.edu.cn/) (26). An intersection analysis of predicted target genes of miR-30b-5p or miR-30e-5p and differential genes in mRNA array was carried out using the Venn diagrams web tool (http://bioinformatics.psb.ugent.be/webtools/Venn/). Moreover, top 20 of functional enrichment analysis was conducted through the Database for Annotation, Visualization and Integrated Discovery (DAVID) v6.8 (https://david.ncifcrf.gov/). The data were further developed by using the cluster Profiler package in R (version 3.6.1).

### Statistical Analysis

Data are expressed as mean ± SD, and analyzed using SPSS 19.0. The differences between groups were analyzed using one-way ANOVA or non-paired *t*-test. All statistical analyses were performed using GraphPad Prism 8 (GraphPad Software, Inc.), and *p* < 0.05 was considered as statistically significant.

## Results

### Macroscopic and Microscopic Appearance of Rat Tumors

After 36 weeks, 17 rats with tongue tumors survived, and 3 rats died of pneumonia or gastroenteritis in the 4NQO induced group. In the control group, all 20 rats survived, and none exhibited tongue tumor formation. Based on macroscopic appearance, the tumors, which were consistently found on the dorsum of the tongue base, presented an exophytic neoplasm, while the normal mucosa was ruddy and smooth (**Figure 1B**). Histopathologically, the tumors spread into the submucosa and underlying muscle layer, forming small nests of atypical keratinizing epithelial cells that surrounded centrally located keratin pearls. No histopathological changes in epithelial cells were observed in the control group (**Figure 1C**).

### miRNA and mRNA Expression Profiles in SD Rats With HNSCC

The expression levels of miR-30 family members (miR-30a-5p, miR-30a-3p, miR-30b-5p, miR-30c-3p, miR-30d-3p, miR-30e-5p and miR-30e-3p) were depressed in tongue SCC tissues (**Table S2**). The expression of the miRNAs miR-30c-5p, miR-30d-5p, and miR-30e-3p was verified to be decreased in tongue SCC tissues, which was in agreement with the results of miRNA microarray analysis (**Figure S1B**). Among the 8799 genes/ESTs analyzed, 372 genes were found to be differentially expressed in tongue SCC tumors (**Table S3**). The genes Ubadc1 (UBA domain containing 1), Ldhb (lactate dehydrogenase B), and Cabc1 (chaperone, ABC1 activity of bc1 complex homolog) were chosen for validation, and the results were consistent with the results of mRNA microarray analysis (**Figure S1A**).

The miRNA-mRNA interaction networks and top 20 enriched pathways suggested that oxidation-reduction might play a vital role in tongue SCC in SD rats (**Figure 1D** and **S1C**). There were 38 miRNAs related to oxidation reduction, including all members of the miR-30 family (**Table S4**). In addition, ion transport and carbohydrate metabolism might have been related to the development of tongue SCC in SD rats (**Figure 1D**).

### The Expression of miR-30 Family Members Was Downregulated in HNSCC

**Figure 2:**
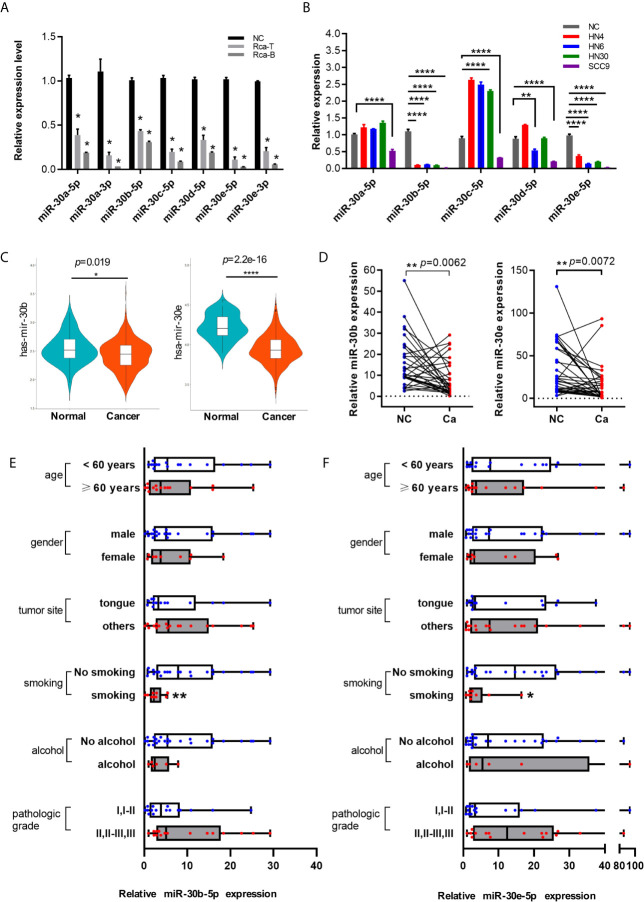
The expression of miR-30 family was downregulated in HNSCC. **(A)** The relative expression level of miR-30 family in rat oral squamous cell lines. **(B)** The expression level of miR-30 family in HNSCC cell lines. **(C)** The expression tendency of miR-30b-5p and miR-30e-5p in HNSCC samples base on TCGA database. **(D)** The expression level of miR-30e-5p and miR-30b-5p in paired HNSCC tissues. **(E, F)** The relative expression level of miR-30b-5p or miR-30e-5p in oral tissues classified according to clinical data (age, gender, tumor site, smoking, alcohol, pathological grade). (*p<0.05, **p<0.01, ****p<0.0001).

The expression levels of miR-30 family members were measured in the SD rat oral squamous cell carcinoma cell lines Rca-T and Rca-B and primary oral mucosal epithelial cells from normal SD rats. As shown in **Figure 2A**, the expression levels of all the miR-30 family members were decreased significantly in Rca-T and Rca-B cells compared with normal SD rat oral mucosal epithelial cells. Similarly, the expression of miR-30 family members was shown to be downregulated in human HNSCC cell lines (HN4, HN6, HN30 and SCC9) (**Figure 2B**). Furthermore, miR-30b-5p and miR-30e-5p expression was significantly downregulated in human HNSCC tissues (**Figures 2C, D**). We found that the expression of miR-30b-5p and miR-30e-5p was lower in samples from the smoking group compared to those from the control group (**Figures 2E, F**).

### The Proliferation and Migration of HNSCC Cells Were Decreased by miR-30b-5p or miR-30e-5p

**Figure 3:**
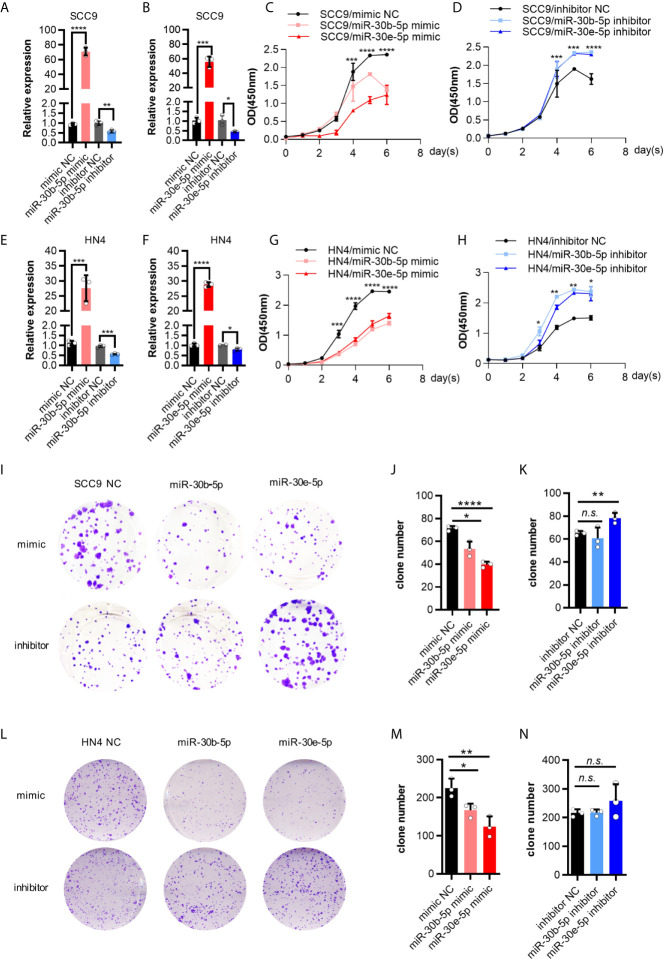
The proliferation of SCC9 and HN4 cells was depressed by miR-30b-5p or miR-30e-5p. **(A)** The expression level of miR-30b-5p in SCC9 cells after transfection with miR-30b-5p mimic or inhibitor. **(B)** The expression level of miR-30e-5p in SCC9 cells after transfection with miR-30e-5p mimic or inhibitor. **(C)** The proliferation of SCC9 cells after transfection with miR-30b-5p or miR-30e-5p mimic. **(D)** The proliferation of SCC9 cells after transfection with miR-30b-5p or miR-30e-5p inhibitor. **(E)** The expression level of miR-30b-5p in HN4 cells after transfection with miR-30b-5p mimic or inhibitor. **(F)** The expression level of miR-30e-5p in HN4 cells after transfection with miR-30e-5p mimic or inhibitor. **(G)** The proliferation of HN4 cells after transfection with miR-30b-5p or miR-30e-5p mimic. **(H)** The proliferation of HN4 cells after transfection with miR-30b-5p or miR-30e-5p inhibitor. **(I–K)** The colony formation of SCC9 cells depressed by miR-30b-5p or miR-30e-5p. **(L–N)** The colony formation of HN4 cells depressed by miR-30b-5p or miR-30e-5p. (*p<0.05, **p<0.01, ***p<0.001, ****p<0.0001, n.s., no significance).

**Figure 4:**
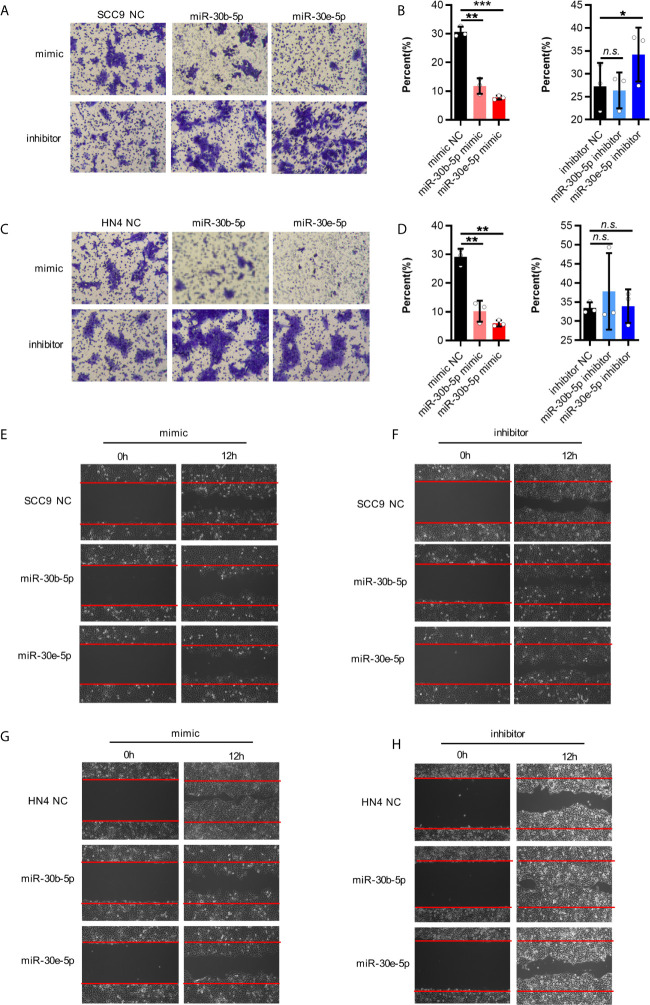
The migration of SCC9 and HN4 cells were depressed by miR-30b-5p or miR-30e-5p. **(A, B)** MiR-30b-5p or miR-30e-5p influenced SCC9 cell migration. **(C, D)** MiR-30b-5p or miR-30e-5p influenced HN4 cell migration. **(E, F)** MiR-30b-5p or miR-30e-5p influenced the rate of SCC9 cell wound closure. **(G, H)** MiR-30b-5p or miR-30e-5p influenced the rate of HN4 cell wound closure. (*p<0.05, **p<0.01, ***p<0.001, n.s., no significance).

First, the expression of miR-30b-5p and miR-30e-5p in SCC9 and HN4 cells was increased after mimic transfection and decreased following inhibitor transfection (**Figures 3A, B, E, F**). After transfection of miR-30b-5p or miR-30e-5p mimic, the proliferation of SCC9 and HN4 cells was decreased (**Figures 3C, G**), while the proliferation of SCC9 and HN4 cells transfected with miR-30b-5p or miR-30e-5p inhibitor was increased (**Figures 3D, H**). The results showed that the colony-forming ability of SCC9 and HN4 cells treated with miR-30e-5p mimic was significantly worse than that of nontreated cells. In contrast, the colony-forming ability of SCC9 and HN4 cells treated with miR-30e-5p inhibitor was significantly better than that of non-treated cells. However, the colony-forming ability of HN4 cells treated with miR-30b-5p or miR-30e-5p inhibitor was not significantly different from that of nontreated cells (**Figures 3I–N**). The results of the transwell migration and wound healing assays suggested that the migration of SCC9 and HN4 cells was decreased by miR-30b-5p or miR-30e-5p (**Figure 4**).

### The Possible Target Genes of miR-30b-5p and miR-30e-5p

**Figure 5:**
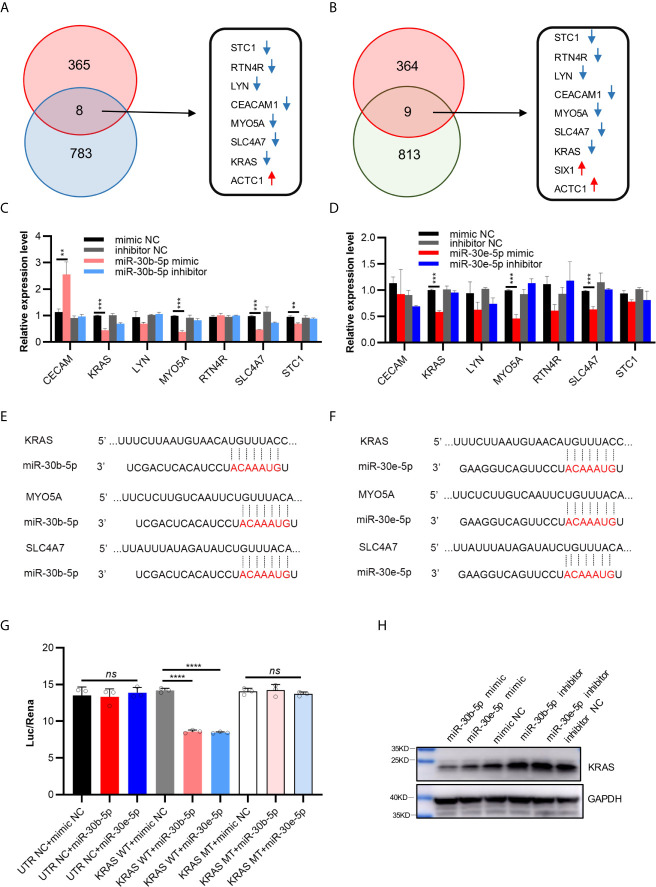
Possible target genes of miR-30b-5p or miR-30e-5p in HNSCC. **(A)** The intersection of differential genes in mRNA microarray (373) and predicted targeted genes of miR-30b-5p by ENCORI (791). There were 8 genes in the intersection, the red arrows representing up-regulated genes and the blue arrows representing down-regulated genes. **(B)** The intersection of differential genes in mRNA microarray (373) and predicted potential targeted genes of miR-30e-5p by ENCORI (822). There were 9 genes in the intersection, the red arrows representing up-regulated genes and the blue arrows representing down-regulated genes. **(C)** The relative expression level of possible target genes in transfected SCC9 with miR-30b-5p mimic or inhibitor. **(D)** The relative expression level of target genes in transfected SCC9 with miR-30e-5p mimic or inhibitor. **(E, F)** Base pairing of miR-30b-5p or miR-30e-5p with 3’ UTR of target mRNAs was predicted by TargetScan (http://www.targetscan.org/vert_72/). **(G)** Relative luciferase activity after cotransfection of 293T cells with miR-30b-5p or miR-30e-5p and vectors containing wilt-type KRAS 3’ -UTR or 3’-mutant KRAS UTR cloned behind a Renilla luciferase reportor gene. **(H)** The expression of KRAS protein after transfection with miR-30b-5p or miR-30e-5p mimic or inhibitor. (**p<0.01, ***p<0.001, ****p<0.0001, n.s., no significance).

A total of 791 genes were predicted to be targets of miR-30b-5p by more than four websites (PITA, RNA22, miRmap microT, miRanda, PicTar, TargetScan), and 822 genes were predicted to be targets of miR-30e-5p by more than four websites (**Tables S5** and **S6**). Intersection of the differentially expressed genes identified by mRNA microarray analysis and the target genes of miR-30b-5p predicted by ENCORI led to the identification of 8 genes (**Figure 5A**). A total of 9 genes were identified by intersection of these data for miR-30e-5p (**Figure 5B**). Among these genes, the expression of CECAM, KRAS, LYN, MYO5A, RTN4R, SLC4A7 and STC1 was downregulated in the mRNA microarray analysis. The differential expression of these genes was validated by real-time PCR in SCC9 and HN4 cells. The expression of KRAS, MYO5A and SLC4A7 was downregulated in both SCC9 and HN4 cells after transfection with miR-30b-5p mimic. Furthermore, the expression of KRAS, MYO5A and SLC4A7 was downregulated by miR-30b-5p mimic (**Figures 5C, D** and **Figures S2A, B**). Base pairing of miR-30b-5p or miR-30e-5p with the 3’ UTR of target mRNAs (KRAS, MYO5A and SLC4A7) was predicted by TargetScan (**Figures 5E, F**). In TCGA HNSCC samples, miR-30b expression was negatively correlated with KARS, MYO5A and SLC4A7 expression. MiR-30e expression had a negative correlation with MYO5A expression but a weak correlation with KRAS and SLC4A7 expression (**Figures S2C–H**). The results of the luciferase assay and western blot analysis revealed that the expression of KRAS could be inhibited by miR-30b-5p or miR-30e-5p, which indicates that KRAS might be the target gene of miR-30b-5p or miR-30e-5p (**Figures 5G, H**).

### Tumor Growth Might Be Suppressed by miR-30b-5p or miR-30e-5p

**Figure 6:**
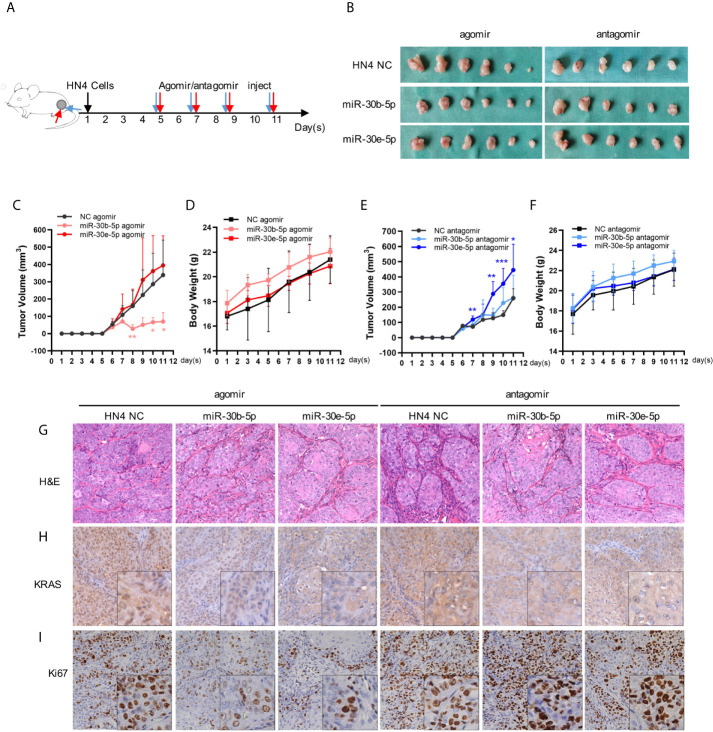
The tumor growth might be suppressed by miR-30b-5p or miR-30e-5p. **(A)** Injection protocols of the miR-30b-5p or miR-30e-5p for subcutaneous tumorigenesis in nude mice. **(B)** Macro images of subcutaneous tumors. **(C, E)** The volume of subcutaneous tumors in nude mice. **(D, F)** The body weight of tumorigenesis nude mice. **(G)** Representative images of H&E staining of subcutaneous tumors injected with different miRNAs (400×). **(H, I)** The expression of KRAS or Ki67 in subcutaneous injected with different miRNAs (400×). (**p<0.01, ***p<0.001).

Subcutaneous tumors were suppressed by miR-30b-5p agomir but promoted by the miR-30e-5p antagomir. The miR-30b-5p antagomir or miR-30e-5p agomir had little influence on tumor growth. Furthermore, no significant weight loss was observed in mice after miRNA injection, indicating that the miRNA had no toxic effects on mice (**Figures 6B–F**). H&E staining of tumors revealed obvious tumor features: large and hyperchromatic nuclei and mitosis were visible, but there was no obvious difference in these features between the six groups (**Figure 6G**). The expression of KRAS and Ki67 in tumors treated with miRNA agomir was weaker than that in tumor treated with the NC, indicating that KRAS expression might be decreased by miR-30b-5p or miR-30e-5p (**Figures 6H, I**).

## Discussion

The development of tobacco-induced head and neck carcinogenesis is a complicated process that is still not completely understood at the molecular level. In the present study, we used a rat model of 4NQO-induced carcinogenesis to identify miRNAs and their targets that are involved in regulating the onset of HNSCC caused by tobacco and to initially identify the key miRNAs contributing to gene regulation during this developmental period.

4NQO exerts potent intracellular oxidative stress effects, and its metabolic product binds to DNA predominantly at guanine residues, which appear to be similar to the damage imposed by the carcinogens present in tobacco (27). Furthermore, this model is similar to human oral carcinogenesis at both the histological and molecular levels (15, 16). In this study, no tumors were observed in the digestive tracts, lungs, or livers of the 4NQO-treated rats, which further confirmed that this rat model simulates aspects of human oral cavity carcinogenesis.

In this study, microarray analysis revealed a set of differentially expressed miRNAs and mRNAs between tumor and normal tissues. These results agree with those of previous studies. For example, miR-21 was found to be oncogenic in HNSCC (28); miR-100 expression is decreased in oral cancer (29); and CDK6 is highly expressed in HNSCC (30), while S100 calcium binding protein B (S100B) expression is downregulated during carcinogenesis (31). These results indicate that the regulatory mechanisms mediated by common miRNAs may play a fundamental role in carcinogenesis. However, more aberrantly expressed miRNAs and mRNAs were found in this study due to the different platforms and methods used as well as the different miRNA and mRNA probe sources. The functions of most of the miRNAs and mRNAs identified in this study were not clear. The functions of these miRNAs and mRNAs will be studied in the future.

Furthermore, the miRNA-gene interaction networks based on the differential miRNA and mRNA expression data revealed some critical members during carcinogenesis. Targets of the downregulated miRNAs (i.e., rno-miR-30a and rno-miR-133a) and targets of the upregulated miRNAs (i.e., rno-miR-21, rno-miR-31, and rno-miR-188) share several target proteins, which indicates that the regulation of these proteins may be complex *in vivo*. This could explain why the expression levels of several targets did not always correlate with the modulation of miRNA levels. In addition, the five key miRNAs belonging to the miR-30 family, namely, rno-miR-30a, rno-miR-30b-5p, rno-miR-30c, rno-miR-30d and rno-miR-30e, were mainly enriched in the oxidation-reduction process. This result is not surprising because 4NQO has been shown to have similar effects as tobacco use, which potently induces intracellular oxidative stress (16). Moreover, miR-30 family members (miR-30b-5p, miR-30e-5p, miR-30d-5p, and miR-30c-5p) were noted to have the highest ratio and enrichment in the miRNA-GO network, and microRNA-GO network analysis revealed that the miR-30 family likely has a critical role in tobacco-related HNSCC.

The miR-30 family includes miR-30a, miR-30b, miR-30c, miR-30d and miR-30e. They all contain the same “seed sequence” in their 5’ termini and are abundantly expressed in the heart under physiological conditions (32). The modulation of miR-30, as a “hub” for the miRNA oncogenesis signal network in solid tumors, has profound impacts on tumorigenesis (33). Volinia et al. studied miRNA profiles in 4419 human samples (3312 neoplastic, 1107 nonmalignant), corresponding to 50 normal tissues and 51 cancer tissues, including epidermal SCC tissues (34). They found that miR-30 expression was downregulated and that this miRNA was physically altered at the DNA copy number level in cancer tissues. MiR-30 family members were also identified as tumor suppressors in a subset of patients with head and neck squamous cell carcinoma (35). Braun et al. discovered that downregulation of miR-30 expression directs the mesenchymal-epithelial transition and invasive potential of anaplastic thyroid carcinomas (36). Downmodulation of miR-30 expression is also found in a variety of tumors. Low expression of miR-30 family members contributes to the development of non-small-cell lung cancer (37), colorectal cancer (38), bladder cancer (39), and so on. In addition, the expression levels of miR-30 members are lower in the lungs of rats exposed to cigarette smoke than those of rats not exposed to cigarette smoke, which indicates that a change in miR-30 family member expression is an early event following exposure to cigarette smoke (40). In this study, we found that miR-30e-5p and miR-30b-5p can inhibit the proliferation, adhesion and migration of HNSCC cells lines. We also observed that miR-30b-5p and miR-30e-5p displayed significantly decreased expression in TCGA HNSCC specimens and that miR-30b-5p and miR-30e-5p expression was associated with tobacco abuse, indicating that miR30-miR-30b-5p and miR-30e-5p may play an important role in HNSCC development caused by tobacco. Moreover, it has been reported that miR-30e-5p suppresses cell proliferation and migration in nasopharyngeal carcinoma (41) and bladder cancer (42). In human hepatocellular carcinoma tissues and cell lines, miR-30b-5p expression is significantly downregulated, and this miRNA mediates DNMT3A to repress proliferation (43). It can also regulate renal cell carcinoma cell proliferation and metastasis through downregulation of GNA13 expression (44). These data were consistent with our studies showing that lower expression of miR-30e-5p and miR-30b-5p inhibited cell proliferation and migration in tumor tissues.

However, miR-30 reported as a “dual” miRNA, with opposite functions in many tumors, including non–small cell lung cancer and pancreatic cancer. MiR-30 family members also act as oncogenic miRNAs in the tumorigenesis of some cancers, indicating that their biological functions are complex. For example, MiR-30e expression is significantly upregulated in plasma samples from the malignant salivary gland tumor group compared to those from the benign group (45). Wang et al. revealed that upregulation of miR-30a expression contributed to tumor formation by inhibiting the expression of forkhead box protein L2 (FOXL2) in human granulosa COV43 cells (46). Moreover, miR-30a was overexpressed in the urine of ovarian serous adenocarcinoma patients (47). Gaziel-Sovran A et al. discovered that ectopic expression of miR-30b/30d promoted the metastatic behavior of melanoma cells by directly targeting the GalNAc transferase GALNT7 (48). Recent studies reported that the “dual” role of miR-30e within the same tumor type highlighting opposite effects based on different molecular backgrounds and in particular to *TP53* status (49). Tobacco-associated cancers are generally characterized by high mutation frequencies and definitely have different molecular genetic background from other oral cancers, while 4NQO as a DNA adduct-forming agent can sufficiently mimic the tobacco carcinogenic signature and act as a tobacco-mimetic promoting *TP53* mutation. It might explain why miR-30e-5p significantly downregulated in HNSCC samples but seemed have no effect in xenograft tumor models, which may due to the different genetic background of cell lines. Moreover, miR-30e may have other mechanisms in different tumor types. To the best of our knowledge, the expression of miR-30 in squamous cell carcinoma of epithelial origin is decreased, while in malignant tumors of nonepithelial origin is increased. The target genes of miR-30 in these two kinds of tumors may be different.

To identify which mRNAs are targeted by miR-30b-5p and miR-30e-5p in HNSCC, we predicted the target genes with ENCORI (The Encyclopedia of RNA Interactomes). Candidate targets were further identified from data on upregulated mRNAs in 4NQO-induced tumor specimens. Among these predicted miR-30b-5p and miR-30e-5p targets, *KRAS, MYO5A* and *SLC4A7* were also validated to be altered in different HNSCC cell lines, in which *KRAS* was further validated as the target genes. Remarkably, the *KRAS* gene is the most frequently mutated oncogene in human cancer. Mutant KRAS proteins are insensitive to GAP-induced GTP hydrolysis, which is linked to many aspects of tumor initiation and progression, including deregulation of key signal transduction pathways, altered metabolism, metastasis and drug resistance (50). KRAS mainly activates the PI3K/AKT/mTOR and BRAF/MEK/ERK pathways and activates inflammatory pathways such as the NFκB signaling pathway, which is required for tumor maintenance and malignant transformation (50). Furthermore, studies of KRAS-driven metabolism, including glycolysis, mitochondrial respiration, glutamine metabolism, and pyrimidine metabolism, have identified numerous potential lethal synthetic vulnerabilities that may be associated with a variety of tobacco-induced oncogenic stresses, such as DNA damage and oxidative stress (51). On the other hand, MYO5A and SLC4A7 were also reported to promote proliferation, invasion, metastasis and apoptosis resistance in many cancers (52, 53). Together, our data suggested that decreased miR-30b-5p and miR-30e-5p expression which may target the *KRAS* gene, are implicated in regulating the onset of HNSCC caused by tobacco and promoting the malignant phenotype.

In summary, our studies revealed that lower expression of miR-30 family members, which are part of miRNA-gene regulatory networks and miRNA-GO networks, is probably essential for the development of tobacco-related HNSCC. Targeting of KRAS by miR-30b-5p or miR-30e-5p inhibits cell growth, proliferation and migration in the context of HNSCC, which suggests the potential of miRNA-based therapeutics or the combination of simultaneous targeting of multiple mRNAs with tumor-targeted therapies or resistance mitigation strategies. Taken together, these findings support the role of miR-30b-5p and miR-30e-5p as potential biomarkers and therapeutic agents for tobacco-related HNSCC.

## Data Availability Statement

The original contributions presented in the study are included in the article/**Supplementary Materials**, further inquiries can be directed to the corresponding authors.

## Ethics Statement

The studies involving human participants were reviewed and approved by The Research Ethics Committee of Ninth People’s Hospital, Shanghai Jiao Tong University School of Medicine. The patients/participants provided their written informed consent to participate in this study. The animal study was reviewed and approved by The Experimental Animal Administrative Committee of Shanghai.

## Author Contributions

WC and MY designed the study. TZ and XZ drafted the manuscript. ZZ analyzed microarray data. TZ and QS participated in tissue collection and tissue sample analysis. XZ, XQ, QS and YF did in-vitro and *in vivo* experimental process. ZZ and XZ performed statistical analyses. WC and MY revised and finalized the manuscript. All authors contributed to the article and approved the submitted version.

## Funding

This research was supported by National Natural Science Foundation of China (81874126, 81672829), the National Key Research and Development Program of China (2016YFC0902700), Science and Technology Commission of Shanghai Municipality (16431903300 and 18DZ2291500).

## Conflict of Interest

The authors declare that the research was conducted in the absence of any commercial or financial relationships that could be construed as a potential conflict of interest.

## Supplementary Material

Supplementary Figure 1 Bioinformatic analysis of miRNA and mRNA microarray. **(A)** The relative expression level of Ubadc1, Ldhb, Cabc1 in SD rat tongue SCC. **(B)** The relative expression level of miR-30c-5p, miR-30d-5p, miR-30e-3p in SD rat tongue SCC. **(C)** MicroRNA-gene-network. Red box nodes represent microRNA, and grey cycle nodes represent mRNA. Edges describe the inhibitive effect of microRNA on mRNA. **(D)** MicroRNA-GO-network. Red box nodes represent microRNA, and grey cycle nodes represent GO. Edges describe the effect of microRNA on GO. (***p*<0.01, ****p*<0.001, *****p*<0.0001).

Supplementary Figure 2 Possible target genes of miR-30b-5p or miR-30e-5p in HNSCC. **(A)** The relative expression level of possible target genes in HN4 after transfection with miR-30b-5p mimic or inhibitor. **(B)** The relative expression level of target genes in HN4 after transfection with miR-30e-5p mimic or inhibitor. **(C–E)** Correlation between miR-30b-5p and predicted possible genes in HNSCC samples of TCGA. **(F–H)** Correlation between miR-30e-5p and predicted possible genes in HNSCC samples of TCGA. (***p*<0.01, ****p*<0.001).
