# Supplementary figures and images for: Identification and Confirmation of the miR-30 Family as a Potential Central Player in Tobacco-Related Head and Neck Squamous Cell Carcinoma
Source: Front Oncol. 2021 Jul 13;11:616372. doi: 10.3389/fonc.2021.616372 (PMC8315965; doi:10.3389/fonc.2021.616372)

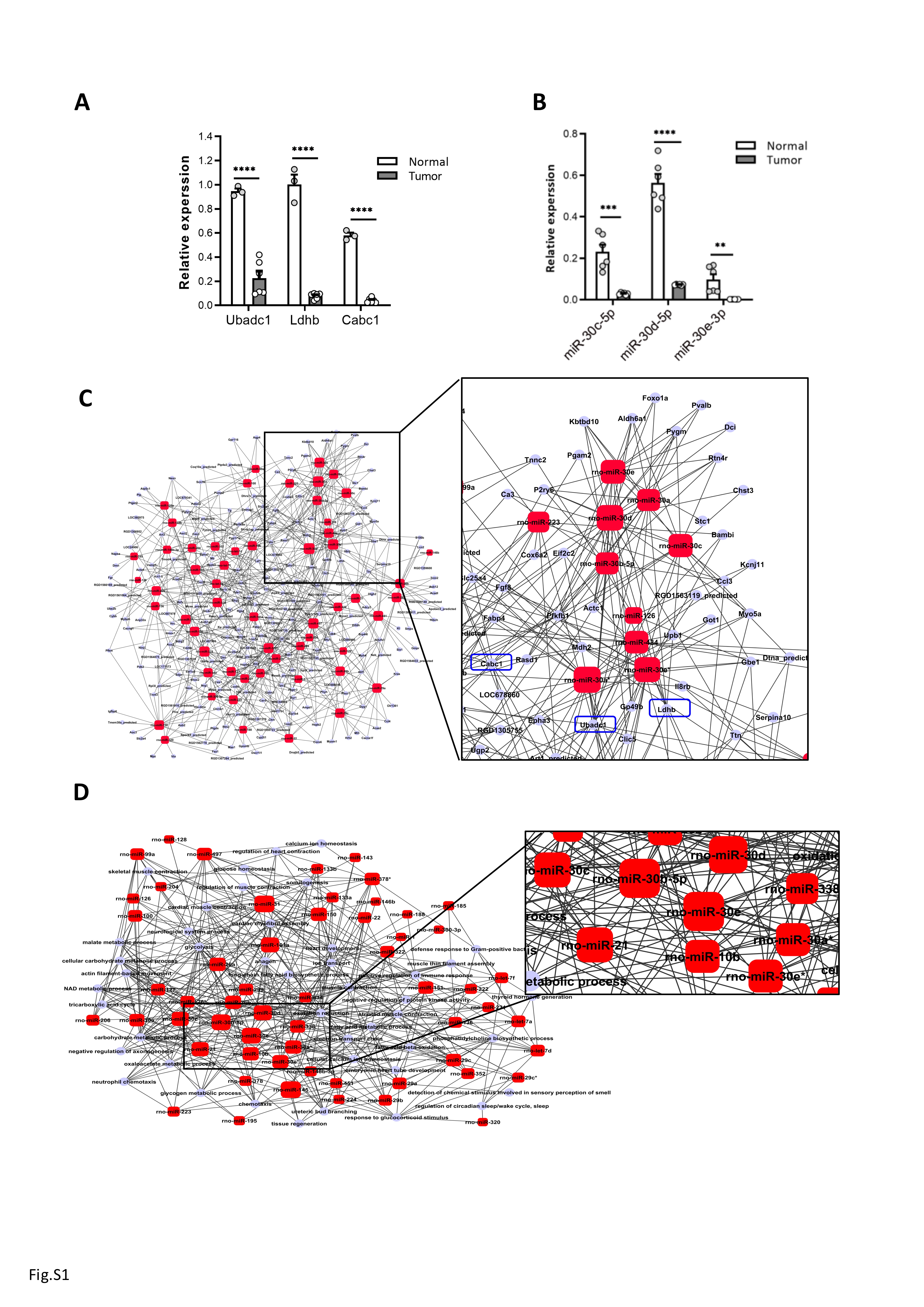

Supplement: Supplementary Figure 1 — Bioinformatic analysis of miRNA and mRNA microarray. (A) The relative expression level of Ubadc1, Ldhb, Cabc1 in SD rat tongue SCC. (B) The relative expression level of miR-30c-5p, miR-30d-5p, miR-30e-3p in SD rat tongue SCC. (C) MicroRNA-gene-network. Red box nodes represent microRNA, and grey cycle nodes represent mRNA. Edges describe the inhibitive effect of microRNA on mRNA. (D) MicroRNA-GO-network. Red box nodes represent microRNA, and grey cycle nodes represent GO. Edges describe the effect of microRNA on GO. (**p<0.01, ***p<0.001, ****p<0.0001). [file Image_1.jpeg]

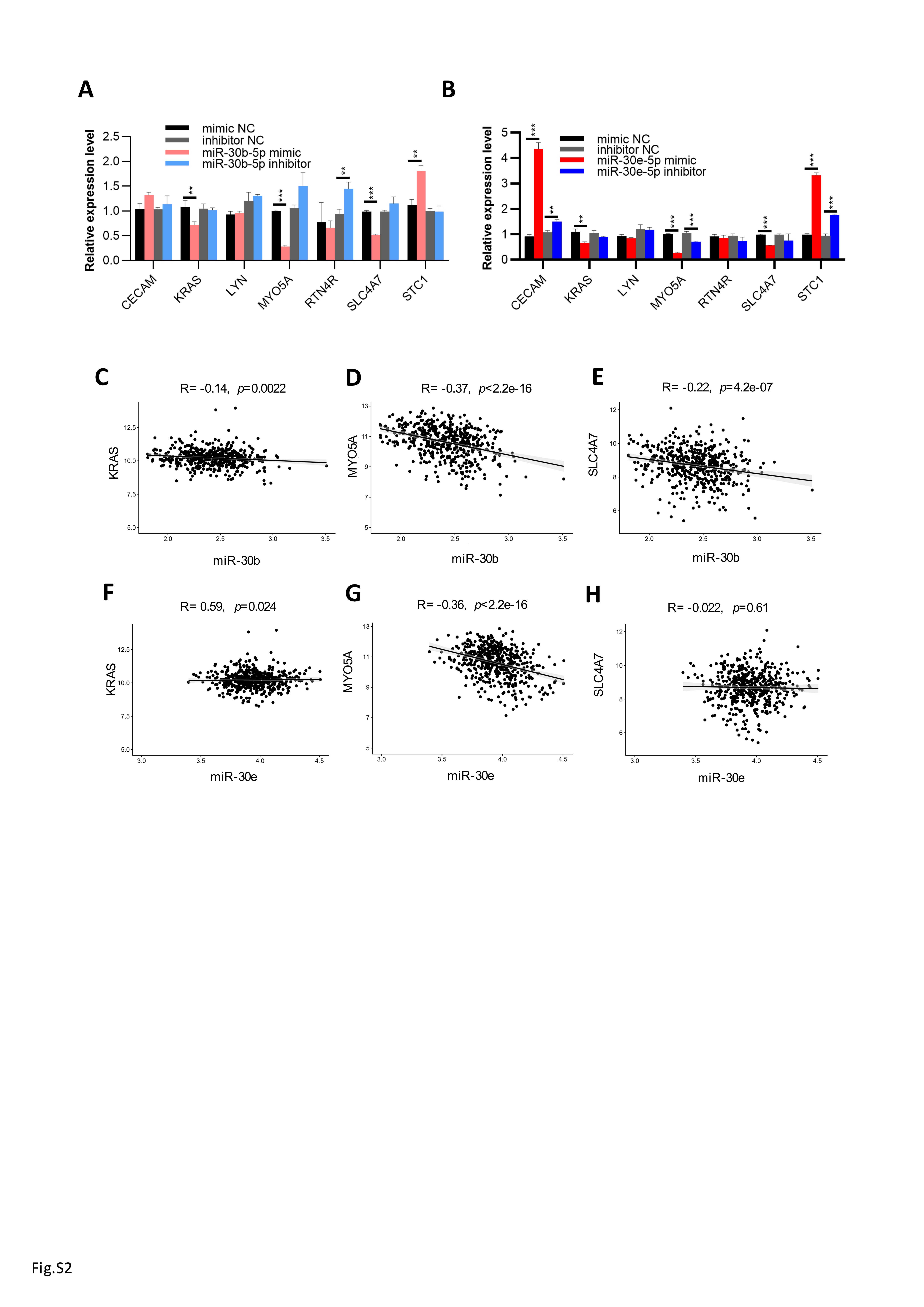

Supplement: Supplementary Figure 2 — Possible target genes of miR-30b-5p or miR-30e-5p in HNSCC. (A) The relative expression level of possible target genes in HN4 after transfection with miR-30b-5p mimic or inhibitor. (B) The relative expression level of target genes in HN4 after transfection with miR-30e-5p mimic or inhibitor. (C–E) Correlation between miR-30b-5p and predicted possible genes in HNSCC samples of TCGA. (F–H) Correlation between miR-30e-5p and predicted possible genes in HNSCC samples of TCGA. (**p<0.01, ***p<0.001). [file Image_2.jpeg]
